# Preoperative Alanine Aminotransferase and Postoperative ALT Elevation: A Retrospective Cohort Study

**DOI:** 10.3390/jcm15176824

**Published:** 2026-09-03

**Authors:** Jisun Choi, Hyun Young Lim, Jeayoun Kim, Seungwon Lee, MiHye Park

**Affiliations:** Department of Anesthesiology and Pain Medicine, Samsung Medical Center, Sungkyunkwan University School of Medicine, 81 Ilwon-ro, Gangnam-gu, Seoul 06351, Republic of Korea

**Keywords:** alanine aminotransferase, postoperative ALT elevation, preoperative assessment, perioperative outcomes, non-cardiac surgery

## Abstract

**Background:** Modestly elevated serum alanine aminotransferase (ALT) levels are associated with increased morbidity and mortality in the general population; however, the perioperative significance of mildly elevated preoperative ALT levels in surgical patients remains unclear. **Methods:** This retrospective cohort study included adults undergoing non-hepatic, non-cardiac surgery at Samsung Medical Center. Patients with preoperative ALT measured within 3 months and <2 times the upper limit of normal (ULN) were eligible. Preoperative ALT was categorized as lower (men 0–34 U/L; women 0–22 U/L) or higher (men 35–82 U/L; women 23–66 U/L). The primary outcome was marked postoperative ALT elevation, operationally defined as any postoperative ALT value > 5 times the institutional ULN within 21 days after surgery. Propensity score matching was performed. Secondary outcomes included long-term ALT elevation, defined as an increase from the preoperative ALT value to >2 times the ULN at the last available measurement within 1 year after surgery, and 1-year all-cause mortality. **Results:** Among 43,892 patients, 7740 (17.6%) were classified into the higher ALT group, and marked postoperative ALT elevation occurred in 733 patients (1.7%). After 1:1 propensity-score matching, 7660 patient pairs were analyzed, and marked postoperative ALT elevation occurred more frequently in the higher than in the lower ALT group (2.7% [204/7660] vs. 1.6% [121/7660]; OR 1.72, 95% CI 1.36–2.16; *p* < 0.001). Higher preoperative ALT was also associated with long-term ALT elevation (OR 4.96, 95% CI 3.65–6.74; *p* < 0.001) and increased 1-year mortality (5.4% vs. 4.4%; OR 1.25, 95% CI 1.08–1.45; *p* = 0.003). **Conclusions:** Higher preoperative ALT was associated with marked postoperative ALT elevation, long-term ALT elevation, and 1-year mortality, even within the conventional normal range. Preoperative ALT may therefore warrant consideration when evaluating postoperative risk.

## 1. Introduction

Postoperative hepatocellular injury and marked aminotransferase elevation may occur after surgery and have been associated with adverse outcomes [1]. The condition typically arises from underlying liver dysfunction, perioperative hypoxia, hypoperfusion, and exposure to hepatotoxic drugs [2,3]. Because the severity of cirrhosis strongly correlates with perioperative risk [3], preoperative screening for underlying liver dysfunction remains an important strategy for reducing the risk of clinically significant postoperative hepatic complications.

Liver chemistries—commonly included in comprehensive metabolic panels—are indirect markers of hepatobiliary disease [4,5]. Serum aminotransferases, including alanine aminotransferase (ALT) and aspartate aminotransferase (AST), leak from injured hepatocytes into the circulation. Among these, serum ALT measurement is widely used to detect liver disease in both the general and surgical populations [4]. Preoperative screening protocols vary across countries and institutions. ALT is commonly included in preoperative biochemical assessment, although the thresholds prompting further evaluation or postponement of surgery vary among institutions and clinical settings [3]. However, the absence of a universal reference range and inter-laboratory variability complicate what is considered “normal” [4]. Recently derived reference intervals from a metabolically and histologically verified Asian cohort suggest sex-specific upper limit of normal (ULN) of 34 U/L for males and 22 U/L for females [6].

Beyond diagnosis, ALT also carries prognostic value across diverse clinical settings. In liver surgery, peak postoperative transaminase levels are widely used as surrogate endpoints of hepatic injury and survival [2,5]. In the general population, such elevations within the normal range correlate with higher risks of morbidity and mortality, both liver-related and non-liver-related [7,8].

Given these observations, we conducted a large, single-center retrospective study to determine whether mildly elevated preoperative ALT concentrations—values traditionally not considered abnormal—are associated with adverse postoperative outcomes. This study is a secondary analysis of a perioperative cohort used in our previous study [9]. Whereas the previous study focused on anesthetic technique, the present study evaluates preoperative ALT concentration as the primary exposure in relation to postoperative ALT elevation and long-term outcomes. We hypothesized that higher preoperative ALT concentrations would be associated with postoperative ALT elevation. The primary outcome was marked postoperative ALT elevation, operationally defined as an ALT level >5 times the ULN within 21 days after surgery, with reference to the American College of Gastroenterology (ACG) biochemical severity classification [4]. Patients were stratified by preoperative ALT into lower (normal range: male 0–34 U/L; female 0–22 U/L) and higher (male 35–82 U/L; female 23–66 U/L) groups, based on proposed Asian reference intervals [6], with one-year outcomes assessed as secondary endpoints.

## 2. Materials and Methods

**Study design and ethical approval**

This single-center retrospective cohort study was a secondary analysis of a previously established perioperative database of adult patients undergoing prolonged general anesthesia at Samsung Medical Center. The source database included patients who underwent general anesthesia for ≥3 h with either sevoflurane-based inhalational anesthesia or propofol-based total intravenous anesthesia (TIVA) between January 2010 and September 2022 [9]. Because the present analysis included outcomes assessed up to 1 year after surgery, the analytic cohort was restricted to patients who underwent surgery through August 2021. Accordingly, 1194 patients who underwent surgery from September 2021 onward and therefore did not have complete 1-year follow-up data were excluded. The final analytic cohort comprised 43,892 patients who underwent surgery between January 2010 and August 2021, with follow-up extending through August 2022. The present study addressed a distinct research question from our previous publication. Whereas the previous study evaluated anesthetic technique in relation to postoperative ALT elevation, the current study evaluated preoperative ALT concentration as the primary exposure and assessed subsequent postoperative ALT elevation and longer-term outcomes. As all data were extracted from the Clinical Data Warehouse (CDW) Darwin-C system, in which personal identifiers are removed before data extraction, the requirement for individual informed consent was waived.

**Patients collection**

Patients who underwent cardiac, transplantation, or hepatobiliary–pancreatic surgery, including liver resection and pancreaticoduodenectomy, were excluded. Preoperative ALT was defined as the most recent value obtained within 3 months before surgery; when multiple measurements were available, the value closest to the date of surgery was used. Patients with preoperative ALT levels > 2 times the institutional ULN were excluded. The institutional reference range for serum ALT is 0–41 U/L for males and 0–33 U/L for females. Pregnant patients, patients who underwent general or regional anesthesia within 21 days before or after the index surgery, and those with missing data for covariates required for propensity-score matching were also excluded [10].

**Data collection**

In this study, 43,892 patients who underwent surgery between January 2010 and August 2021 were included and follow-up extended through August 2022. The following baseline characteristics were collected: age, sex, body mass index, American Society of Anesthesiologists physical status, relevant comorbidities, preoperative laboratory values, duration and type of anesthesia, surgical characteristics, intraoperative hemodynamic variables, transfusion, and perioperative exposure to potentially hepatotoxic medications. The following baseline patient characteristics were collected: age, sex, body mass index (BMI), American Society of Anesthesiologists (ASA) physical status, the presence of underlying diseases (alcohol consumption, smoking, chronic liver disease, diabetes mellitus, hypertension, hyperlipidemia, cerebrovascular accident, chronic obstructive pulmonary disease, coronary artery disease, congestive heart failure), and preoperative laboratory results (ALT, albumin, hemoglobin, fasting glucose, platelet, international normalized ratio and glomerular filtration rate estimate using the Chronic Kidney Disease Epidemiology Collaboration equation [11]). The duration of anesthesia, type of surgery, emergency surgery, department of surgery, intraoperative use of vasoactive drugs (intravenous ephedrine > 5 mg or phenylephrine > 100 µg), intraoperative vasoactive inotropic scores (VIS), the number of intraoperative packed red blood cells, and the frequency of hypotension episodes per hour during surgery, defined as the incidence of mean arterial pressure < 65 mmHg recorded in our electronic vital sign sheets (at 5 min intervals), was extracted. Data regarding the use of commonly prescribed hepatotoxic medications in surgical patients were collected for the period from 24 h before surgery to 24 h after surgery: steroids, acetaminophen, nonsteroidal anti-inflammatory drugs (NSAIDs), antibiotics (amoxicillin/clavulanate, isoniazid, trimethoprim/sulfamethoxazole, fluoroquinolones, macrolides, nitrofurantoin, and minocycline) and antiepileptics (phenytoin, carbamazepine, lamotrigine, and valproate).

**Outcomes**

The primary outcome was marked postoperative ALT elevation, defined as an ALT value > 5 times the institutional ULN within 21 days after surgery. This endpoint reflects biochemical hepatocellular injury and does not necessarily indicate clinically overt hepatic dysfunction or liver failure. The cut-off values for marked ALT elevation were >205 U/L and >165 U/L in male and female, respectively. The threshold of greater than 5 times the ULN was selected with reference to the ACG biochemical severity classification, which classifies ALT elevations as normal (≤ULN), borderline (<2 × ULN), mild (2–5 × ULN), moderate (5–15 × ULN), and severe (>15 × ULN) [4]. In the absence of a validated definition of postoperative liver injury based on ALT elevation, we operationally defined marked postoperative ALT elevation as an ALT level >5 times the institutional ULN, with reference to the ACG biochemical severity classification. Long-term postoperative ALT elevation was defined as an increase in ALT from the preoperative value to >2 times the institutional ULN at the last available measurement between postoperative days 22 and 365. This endpoint was used to identify patients with long-term postoperative ALT elevation during follow-up after the acute postoperative phase. One-year all-cause mortality was ascertained from the hospital patient registry, in which death status is updated through the national healthcare information system.

**Statistical analysis**

No a priori sample size calculation was performed; all eligible patients were included. Continuous variables are presented as means with standard deviations or medians with interquartile ranges, as appropriate. Comparisons between unmatched groups were performed using Student’s *t*-test or the Wilcoxon rank-sum test, as appropriate. For propensity score-matched comparisons, paired *t*-tests or Wilcoxon signed-rank tests were used for continuous variables, and the exact McNemar test was used for binary outcomes. Continuous predictors were examined using unadjusted histograms and categorized when clear non-linearity was observed (Figure S1); selected variables were categorized using clinically relevant cut-offs. Categorical variables are presented as counts (percentages) and compared using the χ^2^ or Fisher’s exact test, as appropriate.

Multivariable linear regression with categorized predictors was used to identify factors associated with postoperative ALT concentrations, with results reported as regression coefficients and 95% confidence intervals (CIs). To evaluate the association between preoperative ALT concentration and the probability of marked postoperative ALT elevation, restricted cubic spline analysis was performed using a multivariable logistic regression model with four knots placed at the 5th, 35th, 65th, and 95th percentiles of preoperative ALT. The model was adjusted for variables independently associated with postoperative ALT in the multivariable linear regression analysis. Overall association and nonlinearity were assessed using likelihood ratio tests. Univariable logistic regression analyses were subsequently performed to identify factors associated with marked postoperative ALT elevation. Variables with *p* < 0.05 in the univariable analyses were included in the multivariable logistic regression model. Results are reported as odds ratios (ORs) with 95% CIs.

For the primary analysis, patients were classified into lower (male, 0–34 U/L; female, 0–22 U/L) and higher (male, 35–82 U/L; female, 23–66 U/L) preoperative ALT groups based on thresholds proposed for metabolically and histologically healthy Asian populations. The groups were matched 1:1 using nearest-neighbor propensity-score matching without replacement, with a caliper width of 0.1 SD of the logit of the propensity score, targeting the average treatment effect in the treated (ATT). Propensity scores were estimated from pre-specified demographic, clinical, hemodynamic, surgical, and anesthetic covariates (variables in Table 1). Covariate balance after matching was assessed using standardized mean differences (SMDs), with values < 0.10 indicating adequate balance. Binary outcomes in the propensity-score-matched cohort were compared using McNemar’s test to account for the 1:1 matched-pair structure, and matched odds ratios with 95% CIs were calculated from discordant pairs. To explore potential differences in outcome ascertainment, we compared the postoperative day on which the maximum ALT value was observed between the propensity score-matched groups using the Wilcoxon signed-rank test. An additional matched analysis evaluated marked postoperative ALT elevation when the maximum observed ALT occurred within postoperative day 7. Matched odds ratios were calculated from discordant pairs, and statistical significance was assessed using the exact McNemar test. For the primary matched analysis of long-term postoperative ALT elevation, only matched pairs in whom both patients had available long-term ALT measurements were included.

To address the potential influence of variables occurring after measurement of the exposure, we performed an additional propensity-score-matching analysis using only covariates available before surgery. Intraoperative and perioperative variables were excluded from this sensitivity model. Patients in the higher and lower ALT groups were matched 1:1 using nearest-neighbor matching without replacement, and covariate balance was assessed using SMDs. Analyses were performed in R 3.6.1 and SPSS 29; two-sided *p* values < 0.05 were considered statistically significant.

## 3. Results

**Study Population**

**Figure 1:**
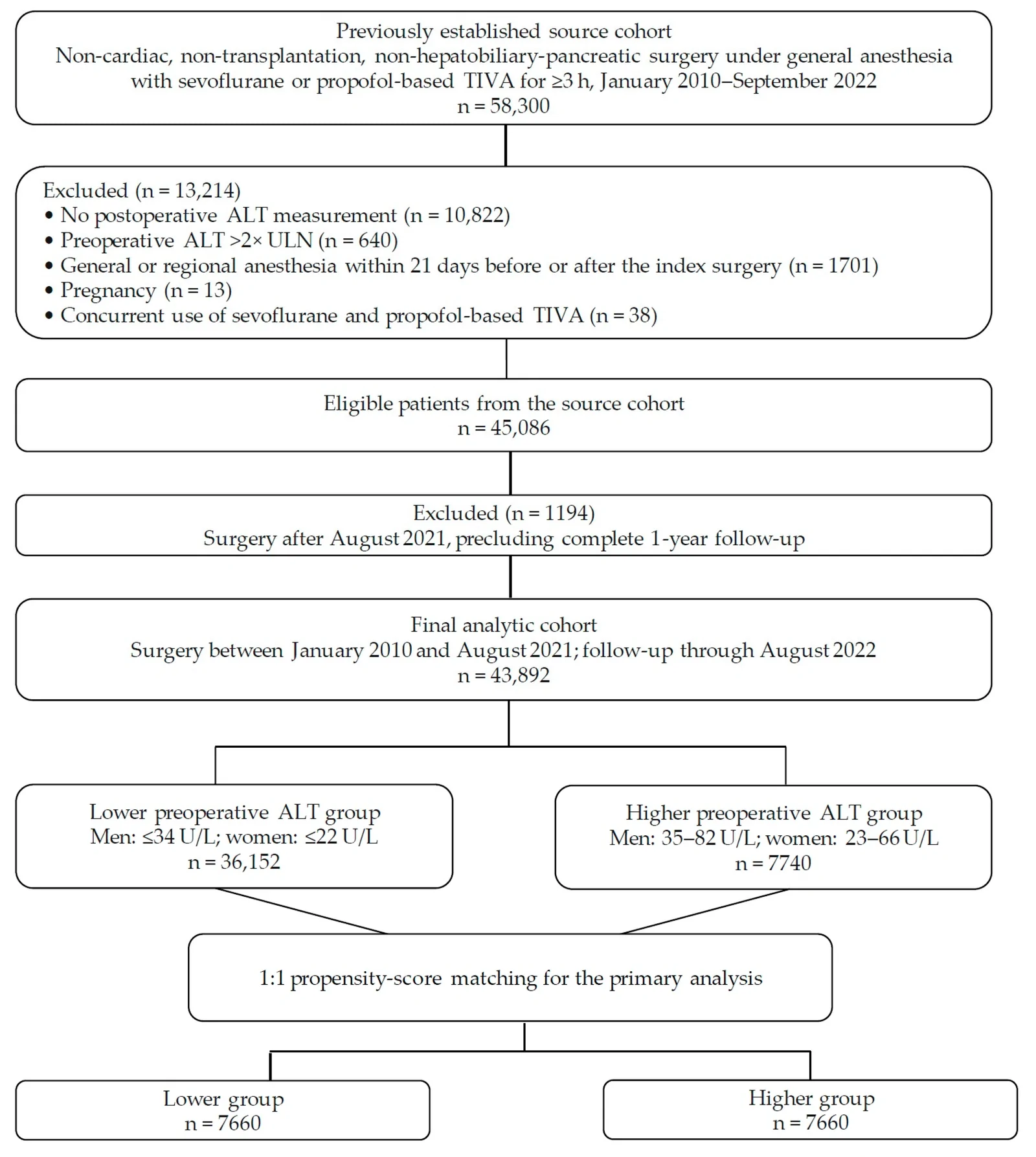
Flow chart of the study cohort. ALT, alanine aminotransferase; ULN, upper limit of normal; TIVA, total intravenous anesthesia.

Of the 58,300 patients in the previously established source cohort, 13,214 were excluded according to the predefined eligibility criteria, leaving 45,086 eligible patients. Because outcomes were assessed for up to 1 year after surgery, an additional 1194 patients who underwent surgery from September 2021 onward and consequently lacked complete 1-year follow-up were excluded. Thus, 43,892 patients who underwent surgery between January 2010 and August 2021 were included in the final analytic cohort (Figure 1). Of these, 36,152 patients (82.4%) were classified into the lower group and 7740 patients (17.6%) into the higher group. As shown in Table 1, patients in the higher group were more frequently male and had higher BMI, a greater prevalence of hypertension and hyperlipidemia, higher preoperative glucose concentrations, more frequent steroid exposure, a higher proportion of neurosurgical procedures, and more frequent use of TIVA.

The higher group was further stratified into 4488 patients (10.2%) with modest elevated ALT within the institutional ULN (males 35–41 U/L; females 23–33 U/L) and 3252 patients (7.4%) with elevated ALT (males 42–82 U/L; females 34–66 U/L). The proportion of patients with preoperative ALT elevation remained stable over the study period (Figure S2).

**Evaluation of the Relationship Between Preoperative and Postoperative ALT**

**Figure 2:**
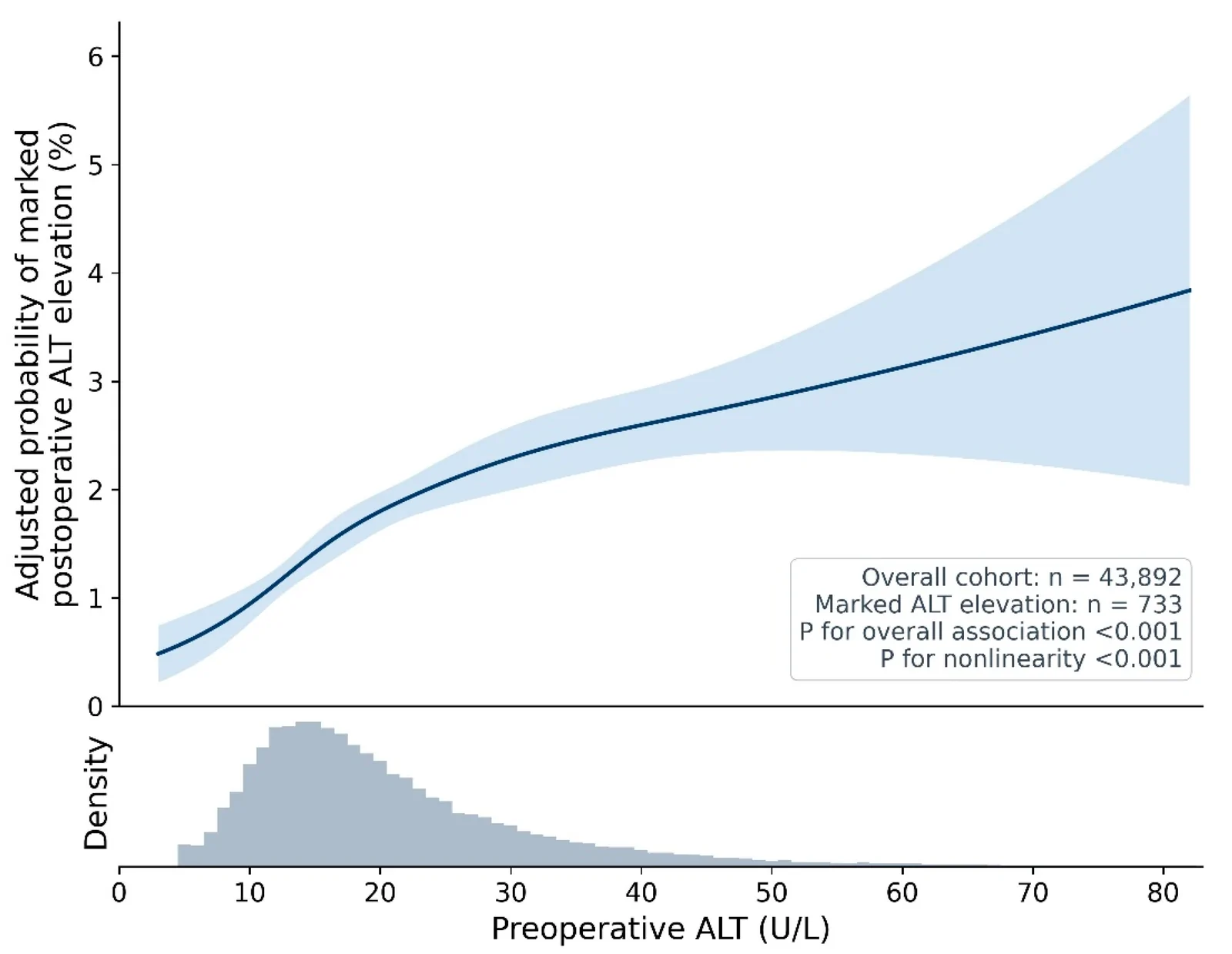
Adjusted probability of marked postoperative ALT elevation according to preoperative ALT concentration. The solid line represents the adjusted predicted probability estimated using a restricted cubic spline model, with the shaded area indicating the 95% confidence interval. The histogram shows the distribution of preoperative ALT concentrations. The model was adjusted for variables independently associated with postoperative ALT in the multivariable analysis presented in Table 2.

Factors associated with postoperative ALT elevation are summarized in Table 2. Higher preoperative ALT, male sex, surgical type, and perioperative drug exposure were independently associated with postoperative ALT. In the overall cohort, restricted cubic spline analysis demonstrated a nonlinear association between preoperative ALT concentration and the adjusted probability of marked postoperative ALT elevation (*p* for overall association < 0.001; *p* for nonlinearity < 0.001), after adjustment for variables independently associated with postoperative ALT in the multivariable analysis presented in Table 2 (Figure 2).

**Association Between Preoperative ALT and Marked Postoperative ALT Elevation**

Overall, marked postoperative ALT elevation occurred in 733 of 43,892 patients (1.7%). For evaluation of the primary outcome, propensity score matching yielded 7660 patients in each group with well-balanced baseline characteristics (Table 1). After propensity-score matching, marked postoperative ALT elevation occurred in 204 of 7660 patients (2.7%) in the higher ALT group and 121 of 7660 patients (1.6%) in the lower ALT group. Among discordant matched pairs, 199 had the outcome only in the higher ALT group and 116 only in the lower ALT group, corresponding to a matched OR of 1.72 (95% CI 1.36–2.16; McNemar *p* < 0.001). Postoperative ALT concentrations were also significantly higher in the higher group (mean difference, 20 U/L; 95% CI, 17–23; *p* < 0.001; Figure S3). In the propensity score-matched cohort, the maximum postoperative ALT value occurred on median postoperative day 2 (IQR, 0–7) in the lower group and postoperative day 1 (IQR, 0–6) in the higher group (*p* < 0.001). Among patients with marked postoperative ALT elevation, the corresponding values were postoperative day 5 (IQR, 2–8) and postoperative day 4.5 (IQR, 2–8), respectively. In an exploratory matched analysis restricted to marked ALT elevation with the maximum observed ALT occurring within postoperative day 7, the outcome occurred in 90 of 7660 patients (1.2%) in the lower group and 151 of 7660 patients (2.0%) in the higher group (matched odds ratio, 1.70; 95% CI, 1.30–2.24; *p* < 0.001).

Subgroup analyses according to the severity of preoperative ALT elevation are presented in Table 3 and demonstrated a clear dose–response relationship with marked postoperative ALT elevation risk. Patients with modest ALT elevations (males 35–41 U/L; females 23–33 U/L) had an OR of 1.69 (95% CI 1.37–2.10; *p* < 0.001), whereas those with elevated ALT (males 42–82 U/L; females 34–66 U/L) had a higher risk of marked postoperative ALT elevation (OR 2.04; 95% CI 1.63–2.56; *p* < 0.001). Postoperative ALT elevation was associated with adverse preoperative characteristics, high-risk surgical features, prolonged anesthesia, greater inotrope or vasopressor requirements, and perioperative steroid use. In contrast, standard-dose acetaminophen (≤4 g/day) and NSAIDs (three times daily) were not associated with postoperative ALT elevation in this study population.

**Long-Term Effects of Preoperative ALT**

Of the 43,892 patients analyzed, 6817 had no available ALT measurements between postoperative days 22 and 365. Among patients with available follow-up measurements, 1.5% (550/37,075) had an increase in ALT from the preoperative value to >2 times the ULN at their last available measurement between postoperative days 22 and 365. Overall 1-year all-cause mortality in the entire cohort was 5.4% (2380/43,892) (Table S1).

In the matched cohort, higher preoperative ALT was associated with substantially increased odds of long-term postoperative ALT elevation and a modest but statistically significant increase in 1-year mortality (Table 4). Patients in the higher group had significantly greater odds of long-term ALT elevation than those in the lower group (matched OR 4.96; 95% CI 3.65–6.74; *p* < 0.001) and higher odds of death at 1 year compared with those in the lower ALT group (OR 1.25; 95% CI 1.08–1.45; *p* = 0.003).

**Sensitivity analysis**

In a sensitivity analysis using only preoperative covariates for propensity-score matching, 7707 matched pairs were identified, with all post-matching absolute SMDs < 0.10 (Table S2). Marked postoperative ALT elevation occurred in 2.7% (207/7707) of the higher ALT group and 1.4% (108/7707) of the lower ALT group (paired OR 1.94; 95% CI, 1.54–2.46; *p* < 0.001). For the long-term analysis, only matched pairs in whom both patients had available ALT follow-up between postoperative days 22 and 365 were included. Among 5029 complete matched pairs, the higher ALT group had significantly greater odds of long-term postoperative ALT elevation than the lower ALT group (paired OR 5.80; 95% CI, 4.17–8.09; *p* < 0.001). One-year all-cause mortality was also higher in the higher ALT group than in the lower ALT group (417/7707 [5.4%] vs. 327/7707 [4.2%]; paired OR 1.30; 95% CI, 1.12–1.51; *p* < 0.001).

## 4. Discussion

In this retrospective cohort study, we examined the association between preoperative ALT concentrations and marked postoperative ALT elevation as well as long-term clinical outcomes following non-hepatic surgery. Although ALT testing is routinely used to detect hepatocellular injury and inform perioperative risk stratification, interpretation remains challenging because universally accepted reference intervals are lacking. To address this limitation, we applied recently proposed ALT thresholds derived from metabolically and histologically healthy Asian populations based on a large living-donor liver database. Using these reference intervals, even modest preoperative ALT elevations were independently associated with a higher risk of early marked postoperative ALT elevation (OR 1.72; 95% CI 1.36–2.16; *p* < 0.001), long-term postoperative ALT elevation at the last available follow-up measurement within 1 year (OR 4.96; 95% CI 3.65–6.74; *p* < 0.001), and increased 1-year all-cause mortality (OR 1.25; 95% CI 1.08–1.45; *p* = 0.003).

Incidental preoperative ALT elevation, defined using conventional reference ranges, has been reported in 6–13% of surgical patients [8,12]. Consistent with these reports, we observed a prevalence of 7.4% using the traditional threshold; however, this increased to 10.2% when a recently proposed healthy reference range was applied. Notably, population-based studies suggest an optimal ALT cut-off of approximately 30 U/L for predicting 1-year mortality in men, implying that lower thresholds may better identify individuals at increased risk [7]. Furthermore, many widely used reference intervals—largely derived from older population cohorts—may not accurately reflect truly normal hepatic function [6,13].

ALT is predominantly localized within hepatocytes, and increased serum activity provides a sensitive marker of hepatocellular injury across a wide range of etiologies, including viral, metabolic, toxic, and ischemic insults [4,14]. Postoperative liver injury has traditionally been attributed to perioperative hypotension, hepatic ischemia, or drug-related toxicity. Our findings suggest that marked postoperative ALT elevation is multifactorial, reflecting the combined effects of adverse preoperative status, high-risk surgical characteristics, prolonged anesthesia, hemodynamic support with inotropes or vasopressors, and perioperative steroid exposure. Notably, perioperative acetaminophen and NSAID exposure was not associated with increased postoperative ALT elevation; however, these observational findings should not be interpreted as evidence of hepatic safety [15,16].

The prognostic significance of preoperative ALT levels in patients without overt liver disease remains incompletely understood. Previous studies, however, have primarily focused on the association between preoperative fibrosis-4 index (FIB-4, combined with age, AST, ALT and platelet count) or AST concentration and postoperative outcomes. Analyses using the US National Surgical Quality Improvement Program (NSQIP) database demonstrated that preoperative AST concentrations > 40 U/L were associated with increased 30-day postoperative mortality [17,18]. In octogenarian patients undergoing emergent general surgery, an elevated preoperative AST level (>40 U/L) independently predicted 1-year cumulative mortality (OR 2.26; 95% CI 1.09–4.70; *p* = 0.029) [19]. Similarly, in patients undergoing major head and neck surgery, elevated AST levels were observed in 10.0% of patients and were associated with a nearly threefold increase in postoperative complications (OR 2.93; 95% CI 1.27–6.77; *p* = 0.012) [20]. The FIB-4 score is associated with postoperative mortality and complications in a population without clinically apparent liver disease [21,22]. Taken together with our findings, these reports suggest that higher preoperative aminotransferase levels—whether ALT or AST—may reflect increased susceptibility to perioperative organ injury or underlying systemic vulnerability. This interpretation is consistent with emerging evidence that even aminotransferase values within the conventional reference range are associated with adverse postoperative outcomes in surgical patients.

Previous studies have shown that even modest ALT elevations in the general population may reflect more serious underlying comorbidities and are associated with increased mortality [7,8,23]. Accordingly, ALT may function as an early biomarker of hepatic metabolic burden or systemic inflammation rather than a specific marker of overt liver disease. Importantly, ALT concentrations within conventional reference ranges have also been associated with transient hepatic stress related to obesity, alcohol consumption, recent vigorous physical activity, medication exposure, non-alcoholic fatty liver disease (NAFLD), and metabolic syndrome [13,24,25,26]. In the Third National Health and Nutrition Examination Survey (NHANES III), higher ALT concentrations were associated with an increased risk of coronary heart disease among non-obese individuals without viral hepatitis or excessive alcohol consumption [26,27]. Lifestyle factors—particularly chronic alcohol use—are also well-established contributors to aminotransferase elevation. Excess mortality in these populations may therefore arise not only from alcohol-related end-organ damage, including alcoholic liver disease, but also from comorbid depression, polysubstance use (including smoking), and accidental injury [26,28,29]. In the present study, patients in the higher group had higher BMI and a greater prevalence of hypertension, hyperlipidemia, and elevated fasting glucose concentrations. In contrast, coronary artery disease was more prevalent in the lower group (3.2% vs. 2.4%, *p* < 0.001). This paradoxical association may reflect a methodological limitation of the study, as preoperative ALT values were capped by design, potentially influencing the observed relationship between ALT and established cardiovascular disease. The association remained robust in a sensitivity analysis in which propensity scores were estimated using only preoperative covariates, suggesting that the primary finding was not dependent on adjustment for intraoperative or perioperative factors.

Nevertheless, several limitations temper the direct translation of these findings into routine clinical practice. In many cases, elective surgery cannot be readily deferred solely because of mildly elevated preoperative ALT levels. Current American College of Gastroenterology guidelines for the evaluation of abnormal liver chemistries recommend that patients with mildly elevated aminotransferases (2–5 times the ULN) be initially assessed for medication-related causes, metabolic dysfunction-associated fatty liver disease, and viral hepatitis [4]. Although a comprehensive diagnostic evaluation for every preoperative ALT elevation is often impractical, selected patients—particularly those with metabolic syndrome or elevated BMI—may benefit from targeted assessment for fatty liver disease, beginning with abdominal ultrasonography. Preoperative ALT values should also be interpreted in the context of the planned surgical procedure, anesthetic technique, and anticipated perioperative drug exposure, which may facilitate individualized perioperative assessment and management.

Several limitations warrant consideration. First, the retrospective design is inherently subject to residual confounding and selection bias. In addition, postoperative ALT testing was not standardized, and individual testing frequency was unavailable; therefore, differential surveillance between the groups may have influenced outcome ascertainment. Second, implementation of revised ALT thresholds would require broad consensus across laboratories and clinical guidelines, particularly given demographic variability—including ethnic differences—that affects ALT distributions. The sex-specific ALT thresholds used in this study were derived from an Asian population and should not be considered universally applicable reference values. ALT distributions may vary according to demographic, metabolic, ethnic, and laboratory characteristics; therefore, external validation is required before these thresholds can be incorporated into broader perioperative risk-assessment strategies. Third, long-term ALT measurements were unavailable in a substantial proportion of patients and were obtained according to clinical care rather than a standardized follow-up protocol. Therefore, missingness may have been related to postoperative clinical status or subsequent treatment, and selection and ascertainment bias cannot be excluded. Long-term ALT findings should therefore be considered exploratory. In addition, non-surgical factors, such as chemotherapy exposure, appeared to be dominant contributors to long-term ALT elevation or mortality within the first postoperative year, potentially attenuating the observed association between perioperative ALT changes and long-term outcomes. Higher preoperative ALT was associated with a modest absolute difference in 1-year all-cause mortality (5.4% vs. 4.4%). Given the observational design and potential for residual confounding, this finding should be interpreted as an association rather than evidence of a causal effect of ALT. Finally, this study did not consider AST or FIB-4, focusing instead on evaluating the independent effect of ALT on the outcomes. In addition, because the primary outcome was based solely on ALT elevation, it should be interpreted as a biochemical hepatocellular injury endpoint rather than clinically meaningful hepatic dysfunction. Postoperative bilirubin, INR, and clinical manifestations of liver failure were not incorporated into the outcome definition.

Mild elevations in preoperative ALT were associated with marked postoperative ALT elevation and adverse 1-year outcomes after non-hepatic, non-cardiac surgery; an association with long-term ALT elevation was also observed among patients with available follow-up measurements. These findings suggest that preoperative ALT may provide additional clinically relevant risk information; however, the present study did not formally evaluate the incremental predictive performance of ALT beyond established perioperative risk models. Further prospective studies are needed to establish actionable thresholds and management strategies.

## Figures and Tables

**Table 1:** Baseline demographic, clinical, and perioperative characteristics of patients categorized into lower (male ≤ 34 U/L; female ≤ 22 U/L) and higher (male 35–82 U/L; female 23–66 U/L) groups.

Variable	All Patients			Matched Patients		
	Lower n = 36,152	Higher n = 7740	SMD	Lower n = 7660	Higher n = 7660	SMD
Department of surgery			0.284			0.020
General surgery	10,012 (27.7)	1635 (21.1)		1613 (21.0)	1632 (21.2)	
Orthopedics	3791 (10.5)	1072 (13.9)		1083 (14.1)	1060 (13.8)	
Neurosurgery	7728 (21.4)	2364 (30.5)		2389 (31.1)	2341 (30.5)	
Gynecology	1280 (3.5)	308 (4.0)		307 (4.0)	305 (4.0)	
Urology	5291 (14.6)	885 (11.4)		862 (11.2)	884 (11.5)	
Thoracic surgery—pulmonary	5369 (14.9)	857 (11.1)		837 (10.9)	856 (11.1)	
Others	2681 (7.4)	619 (8.0)		597 (7.8)	610 (7.9)	
Cancer surgery	28,891 (79.9)	5672 (73.3)	0.157	5636 (73.3)	5645 (73.4)	0.003
Emergency	1761 (4.9)	480 (6.2)	0.058	544 (7.1)	475 (6.2)	0.036
Date of surgery			0.085			0.024
2010.1–2012.6	7155 (19.8)	1359 (17.6)		1349 (17.5)	1358 (17.7)	
2012.7–2014.6	5030 (13.9)	1008 (13.0)		1043 (13.6)	1004 (13.1)	
2014.7–2016.6	5183 (14.3)	1079 (13.9)		1044 (13.6)	1071 (13.9)	
2016.7–2018.6	5937 (16.4)	1331 (17.2)		1295 (16.8)	1321 (17.2)	
2018.7–2020.6	6583 (18.2)	1609 (20.8)		1571 (20.4)	1593 (20.7)	
2020.7–2021.8	6264 (17.3)	1354 (17.5)		1386 (18.0)	1341 (17.4)	
Age; year	58.4 (14.0)	55.8 (13.1)	0.190	55.8 (14.3)	55.9 (13.1)	0.003
Sex, Female	21,668 (59.9)	3674 (47.5)	0.252	3646 (47.4)	3659 (47.6)	0.003
Body mass index; kg/m^2^	24.1 (3.4)	25.8 (4.0)	0.453	25.6 (3.9)	25.7 (3.8)	0.016
ASA physical status			0.081			0.027
1	8458 (23.4)	1638 (21.2)		1627 (21.2)	1632 (21.2)	
2	23,270 (64.4)	5267 (68.0)		5194 (67.6)	5236 (68.1)	
3	4163 (11.5)	788 (10.2)		813 (10.6)	773 (10.1)	
4	240 (0.7)	46 (0.6)		54 (0.7)	46 (0.6)	
5	21 (0.1)	1 (0.0)		0 (0.0)	1 (0.0)	
Liver disease	1805 (5.0)	543 (7.0)	0.085	519 (6.8)	535 (7.0)	0.008
Hypertension	10,747 (29.7)	2493 (32.2)	0.054	2484 (32.3)	2472 (32.2)	0.003
Stroke	891 (2.5)	185 (2.4)	0.005	182 (2.4)	184 (2.4)	0.002
Heart failure	109 (0.3)	15 (0.2)	0.022	15 (0.2)	15 (0.2)	<0.001
Diabetes mellitus	4887 (13.5)	1108 (14.3)	0.023	1069 (14.0)	1087 (14.2)	0.007
Coronary artery disease	1144 (3.2)	189 (2.4)	0.044	174 (2.3)	185 (2.4)	0.009
Chronic obstructive pulmonary disease	1026 (2.8)	124 (1.6)	0.084	123 (1.6)	124 (1.6)	0.001
Current alcohol consumption	7577 (21.0)	1565 (20.2)	0.018	1506 (19.6)	1558 (20.3)	0.017
Current smoking	3162 (8.7)	726 (9.4)	0.022	712 (9.3)	721 (9.4)	0.004
Hyperlipidemia	2091 (5.8)	596 (7.7)	0.076	612 (8.0)	586 (7.6)	0.013
Pre-operative laboratory						
Hemoglobin; g/L	132.65 (18.0)	136.96 (17.7)	0.241	136.4 (16.7)	136.9 (17.7)	0.030
Albumin; g/dL	4.34 (0.42)	4.40 (0.43)	0.133	4.4 (0.4)	4.4 (0.4)	0.029
eGFR; mL/min/1.73 m^2^	100.01 (11.6)	102.44 (10.9)	0.215	102.4 (11.5)	102.4 (10.9)	0.006
Total bilirubin; mg/dL	0.57 (0.35)	0.58 (0.37)	0.007	0.58 (0.34)	0.58 (0.37)	0.001
International normalized ratio	0.99 (0.10)	0.97 (0.09)	0.176	0.97 (0.07)	0.97 (0.09)	0.026
Platelet; U/dL	241.8 (71.4)	244.1 (73.5)	0.031	243.0 (65.8)	243.8 (73.5)	0.012
Fasting glucose; mg/dL	112.2 (29.6)	116.5 (32.2)	0.138	117.1 (34.2)	116.2 (31.8)	0.026
Intravenous general anesthesia	10,182 (28.2)	2758 (35.6)	0.161	2788 (36.3)	2729 (35.5)	0.016
Surgical risk			0.134			0.011
Low	3842 (10.6)	1013 (13.1)		1004 (13.1)	1000 (13.0)	
Intermediate	30,124 (83.3)	6450 (83.3)		6392 (83.1)	6411 (83.4)	
High	2186 (6.0)	277 (3.6)		292 (3.8)	277 (3.6)	
Anesthesia duration; h	4.7 (1.7)	4.7 (1.7)	0.010	4.7 (1.7)	4.7 (1.7)	0.004
Intraoperative hypotension event; events per hour	0.8 (1.4)	0.8 (1.4)	0.018	0.77 (1.36)	0.77 (1.39)	0.001
Intraoperative vasoactive drug use	16,215 (44.9)	3114 (40.2)	0.094	3131 (40.7)	3098 (40.3)	0.009
VIS	0.22 (3.46)	0.22 (3.49)	0.001	0.27 (6.63)	0.22 (3.50)	0.008
Intraoperative fluid infusion; 10 mL	159.97 (91.9)	161.21 (92.6)	0.014	160.0 (87.5)	161.1 (92.6)	0.013
Intraoperative red blood cell; unit	0.22 (0.95)	0.20 (0.86)	0.017	0.2 (0.9)	0.2 (0.9)	0.006
Intraoperative bleeding; 10 mL	30.5 (48.8)	32.0 (47.3)	0.030	32.1 (49.1)	32.0 (47.4)	0.002
Perioperative use						
NSAID use	15,376 (42.5)	3666 (47.4)	0.097	3629 (47.2)	3631 (47.2)	0.001
Acetaminophen use	23,502 (65.0)	5291 (68.4)	0.071	5275 (68.6)	5252 (68.3)	0.006
Steroid use	10,322 (28.6)	2681 (34.6)	0.131	2664 (34.7)	2660 (34.6)	0.001
Antibiotics use	2874 (7.9)	646 (8.3)	0.014	647 (8.4)	637 (8.3)	0.005
Antiepilepsy use	848 (2.3)	187 (2.4)	0.005	182 (2.4)	187 (2.4)	0.004

Values are number (proportion) or mean (standard deviation). SMD, standardized mean difference; ASA, American society of anesthesiology; eGFR, estimated glomerular filtration rate; NSAID, Non-Steroidal Anti-Inflammatory Drug; VIS, Vasoactive-Inotropic Score. VIS = dopamine dose (mcg/kg/min) + dobutamine dose (mcg/kg/min) + 100∙epinephrine dose (mcg/kg/min) + 10∙milrinone dose (mcg/kg/min) + 10,000∙vasopressin dose (unit/kg/min) + 100∙norepinephrine dose (mcg/kg/min).

**Table 2:** Univariable and multivariable linear regression analysis for postoperative alanine aminotransferase in the overall patients (n = 43,892).

Variable	Univariable		Multivariable	
	Coefficient (95%CI)	*p*	Coefficient (95%CI)	*p*
Preoperative ALT; U/L, Ref. F: ≤ 22, M: ≤ 34		<0.001		<0.001
F: 23–33, M: 35–41	9.61 (6.47, 12.75)	<0.001	12.82 (9.67, 15.97)	<0.001
F: 34–66, M: 42–82	29.76 (26.13, 33.40)	<0.001	29.50 (25.88, 33.13)	<0.001
Department of surgery, Ref. General surgery		<0.001		<0.001
Orthopedics	−11.66 (−15.05, −8.28)	<0.001	−1.17 (−5.72, 3.39)	0.616
Neurosurgery	−5.83 (−8.53, −3.13)	<0.001	−8.80 (−13.05, −4.55)	<0.001
Gynecology	−23.12 (−28.43, −17.81)	<0.001	−21.39 (−27.59, −15.18)	<0.001
Urology	−13.94 (−17.06, −10.82)	<0.001	−11.01 (−14.48, −7.54)	<0.001
Thoracic surgery—pulmonary	5.16 (2.04, 8.27)	0.001	2.92 (−0.66, 6.50)	0.110
Others	−20.72 (−24.63, −16.80)	<0.001	−20.48 (−25.11, −15.86)	<0.001
Cancer surgery	10.75 (8.43, 13.07)	<0.001	7.39 (4.39, 10.38)	<0.001
Emergency	18.21 (13.90, 22.52)	<0.001	10.21 (5.75, 14.67)	<0.001
Date of surgery, Ref. 2010.1–2012.6		0.454		
2012.7–2014.6	1.06 (−2.29, 4.40)	0.537		
2014.7–2016.6	−3.43 (−6.74, −0.11)	0.042		
2016.7–2018.6	−3.34 (−6.52, −0.17)	0.039		
2018.7–2020.6	−3.76 (−6.84, −0.68)	0.017		
2020.7–2021.8	−4.83 (−7.97, −1.69)	0.003		
Age; yr, Ref. ≤ 55		0.097		
56–75	0.40 (−1.61, 2.41)	0.693		
>75	−1.75 (−5.42, 1.92)	0.350		
Sex, Male	11.75 (9.83, 13.67)	<0.001	9.69 (7.53,11.85)	<0.001
BMI; kg/m^2^, Ref, 18.5–24.9		0.206		
12.8–18.4	−2.56 (−7.86, 2.73)	0.343		
25–30	0.90 (−1.15, 2.94)	0.391		
>30	4.17 (−0.02, 8.35)	0.051		
ASA physical status ≥ 3	10.73 (7.81, 13.66)	<0.001	7.10 (4.01, 10.19)	<0.001
Liver disease	0.30 (−3.92, 4.52)	0.890		
Hypertension	−0.52 (−2.59, 1.55)	0.622		
Stroke	−1.69 (−7.83, 4.45)	0.590		
Heart failure	0.28 (−17.62, 18.18)			
Diabetes mellitus	−2.84 (−5.61, −0.08)	0.044	−6.97 (−9.93, −4.00)	<0.001
Coronary artery disease	3.89 (−1.65, 9.43)	0.168		
Chronic pulmonary disease	5.33 (−0.62, 11.28)	0.079		
Hyperlipidemia	−0.13 (−4.09, 3.84)	0.950		
Current alcohol use	0.06 (−2.27, 2.40)	0.957		
Current smoking	4.67 (1.33, 8.01)	0.006	1.14 (−2.30,4.58)	0.515
Pre-operative laboratory test				
Hemoglobin; g/L, Ref. F: > 120, M: > 135		<0.001		<0.001
F: ≤ 100–120, M: ≤ 100–135	−2.66 (−4.79, −0.54)	0.014	−5.94 (−8.24, −3.65)	<0.001
80- ≤ 100	−0.51 (−5.28, 4.26)	0.834	−10.85 (−16.10, −5.61)	<0.001
<80	−3.04 (−22.05, 15.97)	0.754	−30.49 (−49.68, −11.30)	0.002
Albumin; g/dL, Ref. ≥4.2		0.999		
<4.2–3.5	1.43 (−0.92, 3.78)	0.232	−0.41 (−2.92, 2.10)	0.747
<3.5	11.40 (6.37, 16.44)	<0.001	−0.84 (−6.56, 4.89)	0.775
eGFR; mL/min/1.73 m^2^, Ref. ≥90		<0.001		<0.001
60- < 90	−0.79 (−3.37, 1.78)	0.546	−1.24 (−3.93, 1.45)	.367
<60	54.26 (34.33, 74.18)	<0.001	47.17 (27.31, 67.03)	<0.001
Total bilirubin; mg/dL, Ref. ≤1.2		0.151		
>1.2- < 2.0	8.29 (2.42, 14.15)	0.006	3.06 (−2.77, 8.89)	0.304
≥2.0	−8.80 (−17.30, −0.29)	0.043	−6.49 (−15.05, 2.07)	0.137
International normalized ratio, Ref. ≤1.2		<0.001		<0.001
>1.2- < 2.0	18.24 (10.95, 25.54)	<0.001	9.00 (1.33, 16.68)	.022
≥2.0	73.85 (49.16, 98.54)	<0.001	65.50 (41.07, 89.94)	<0.001
Platelet; U/dL, Ref. ≥200 K		0.001		0.001
≥100 K, >200 K	0.20 (−1.15, 2.75)	0.420	−0.78 (−2.74, 1.18)	0.369
<100 K	32.78 (22.42, 43.14)	<0.001	18.98 (8.55, 29.41)	<0.001
Fasting glucose; mg/dL, Ref. ≤120		0.003		
121–180	6.50 (4.20, 8.81)	<0.001	3.82 (1.36, 6.28)	0.002
>180	12.71 (7.72, 17.70)	<0.001	5.50 (0.34, 10.66)	0.037
Intravenous GA (vs. inhalation GA)	−4.86 (−6.95, −2.78)	<0.001	−4.20 (−7.14, −1.29)	0.005
Surgical risk, Ref. Low		<0.001		<0.001
Intermediate	13.61 (10.57, 16.64)	<0.001	6.36 (2.99, 9.73)	<0.001
High	35.40 (30.48, 40.31)	<0.001	17.71 (12.08, 23.34)	<0.001
Anesthesia duration; h	1.84 (1.27, 2.40)	<0.001	0.98 (0.09, 1.86)	0.030
Intra-operative hypotension; events	0.32 (−0.36, 1.00)	0.362		
Intraoperative vasoactive drug use	5.07 (3.15, 6.98)	<0.001	1.24 (−0.77, 3.24)	0.226
VIS	1.85 (1.58, 2.13)	<0.001	1.28 (1.00, 1.56)	<0.001
Intra-operative fluid infusion; 10 mL	0.06 (0.05, 0.07)	<0.001	0.04 (0.02, 0.06)	<0.001
Intra-operative red blood cell; unit	4.98 (3.97, 6.00)	<0.001	4.75 (2.89, 6.61)	<0.001
Intra-operative bleeding; 10 mL	0.08 (0.06, 0.10)	<0.001	−0.08 (−0.11, −0.04)	<0.001
Perioperative NSAID use	−0.70 (−2.62, 1.22)	0.475		
Perioperative acetaminophen use	−10.74 (−12.74, −8.74)	<0.001	−7.12 (−9.46, −4.77)	<0.001
Perioperative steroid use	6.89 (4.81, 8.97)	<0.001	11.46 (8.80, 14.13)	<0.001
Perioperative antibiotics use	11.30 (7.81, 14.80)	<0.001	5.23 (1.67, 8.80)	0.004
Perioperative antiepilepsy use	−1.15 (−2.62, 1.22)	0.475		

ASA, American society of anesthesiology; eGFR, estimated glomerular filtration rate; F, female; M, male; NSAID, Non-Steroidal Anti-Inflammatory Drug; VIS, Vasoactive-Inotropic Score.

**Table 3:** Univariable and multivariable logistic regression analyses of marked postoperative ALT elevation in the overall cohort (n = 43,892).

Variable	Univariable		Multivariable	
	Odds Ratio (95%CI)	*p*	Odds Ratio (95%CI)	*p*
Preoperative ALT; U/L, Ref. F: ≤ 22, M: ≤ 34		<0.001		<0.001
F: 23–33, M: 35–41	1.67 (1.35, 2.06)	<0.001	1.69 (1.37, 2.10)	<0.001
F: 34–66, M: 42–82	2.13 (1.71, 2.64)	<0.001	2.04 (1.63, 2.56)	<0.001
Department of surgery, Ref. General surgery		<0.001		<0.001
Orthopedics	0.54 (0.40, 0.73)	<0.001	1.15 (0.78, 1.69)	0.477
Neurosurgery	1.09 (0.91, 1.31)	0.364	1.19 (0.90, 1.59)	0.256
Gynecology	0.33 (0.18, 0.61)	<0.001	0.41 (0.22, 0.78)	0.007
Urology	0.33 (0.24, 0.46)	<0.001	0.46 (0.32, 0.65)	<0.001
Thoracic surgery-pulmonary	1.06 (0.87, 1.32)	0.535	1.30 (1.01, 1.67)	0.044
Others	0.30 (0.19, 0.48)	<0.001	0.35 (0.21, 0.58)	<0.001
Cancer surgery	1.66 (1.35, 2.05)	<0.001	1.58 (1.22, 2.04)	<0.001
Emergency	2.06 (1.61, 2.64)	<0.001	1.49 (1.12, 1.97)	0.006
Date of surgery, Ref. 2010.1–2012.6		0.138		
2012.7–2014.6	0.93 (0.74, 1.18)	0.569		
2014.7–2016.6	0.66 (0.51, 0.86)	0.002		
2016.7–2018.6	0.71 (0.56, 0.91)	0.006		
2018.7–2020.6	0.79 (0.63, 0.99)	0.045		
2020.7–2021.8	0.73 (0.58, 0.93)	0.010		
Age; yr, Ref. ≤55		0.895		
56–75	0.99 (0.85, 1.16)	0.924		
>75	0.84 (0.62, 1.13)	0.251		
Sex, Male	0.91 (0.79, 1.06)	0.222		
Body mass index; kg/m^2^, Ref, 18.5–24.9		0.241		
12.8–18.4	1.50 (1.06, 2.10)	0.021		
25–30	0.95 (0.81, 1.12)	0.541		
>30	1.06 (0.78, 1.45)	0.707		
ASA physical status ≥ 3	1.42 (1.16, 1.73)	<0.001	1.38 (1.11, 1.73)	0.004
Liver disease	0.91 (0.65, 1.28)	0.595		
Hypertension	0.90 (0.76, 1.06)	0.191		
Stroke	1.00 (0.63, 1.61)	0.994		
Heart failure	1.97 (0.72, 5.34)	0.184		
Diabetes mellitus	0.82 (0.65, 1.03)	0.081		
Coronary artery disease	1.08 (0.72, 1.63)	0.706		
Chronic pulmonary disease	0.99 (0.62, 1.57)	0.962		
Hyperlipidemia	1.30 (0.99, 1.71)	0.060		
Current alcohol	0.83 (0.69, 1.01)	0.058		
Current smoking	1.23 (0.97,1.56)	0.087		
Pre-operative laboratory test				
Hemoglobin; g/L, Ref. F: > 120, M: > 135		0.002		0.002
F: ≤ 100–120, M: ≤ 100–135	0.69 (0.57, 0.84)	<0.001	0.73 (0.62, 0.87)	<0.001
80- ≤ 100	0.99 (0.69, 1.41)	0.953	0.74 (0.49, 1.12)	0.151
<80	1.53 (0.48, 4.82)	0.472	0.79 (0.23, 2.65)	0.700
Albumin; g/L, Ref. ≥4.2		0.019		0.029
<4.2–3.5	0.84 (0.70, 1.02)	0.079	0.76 (0.62, 0.94)	0.010
<3.5	1.49 (1.08, 2.05)	0.015	0.81 (0.53, 1.25)	0.346
eGFR; mL/min/1.73 m^2^, Ref. ≥90		0.447		
60- < 90	0.83 (0.67, 1.03)	0.089		
<60	2.39 (0.88, 6.53)	0.088		
Total bilirubin; mg/dL, Ref. ≤1.2		0.726		
>1.2- < 2.0	1.12 (0.73, 1.71)	0.612		
≥2.0	0.86 (0.43, 1.74)	0.679		
International normalized ratio Ref. ≤1.2		0.016		0.018
>1.2- < 2.0	2.32 (1.58, 3.41)	<0.001	1.82 (1.15, 2.88)	0.011
≥2.0	3.96 (1.44, 10.92)	<0.001	2.85 (0.95, 8.51)	0.061
Platelet, U/dL, Ref. ≥200 K		0.225		
≥100 K, >200 K	0.93 (0.80, 1.08)	0.334		
<100 K	2.42 (1.43, 4.09)	0.001		
Fasting glucose; mg/dL, Ref. ≤120		0.028		0.093
121–180	1.52 (1.29, 1.79)	<0.001	1.18 (0.99, 1.40)	0.063
>180	1.42 (1.01, 2.01)	0.046	0.87 (0.60, 1.25)	0.445
Intravenous GA (vs. inhalation GA)	1.14 (0.97, 1.33)	0.104		
Surgical risk, Ref. Low		<0.001		<0.001
Intermediate	2.47 (1.74, 3.49)	<0.001	1.70 (1.18, 2.45)	0.005
High	4.39 (2.92, 6.61)	<0.001	2.56 (1.61, 4.07)	<0.001
Anesthesia duration; h	1.11 (1.07, 1.15)	<0.001	1.12 (1.06, 1.19)	<0.001
Intra-operative hypotension; events	1.03 (0.98, 1.08)	0.215		
Intraoperative vasoactive drug use	1.29 (1.11, 1.49)	<0.001	1.18 (1.01, 1.38)	0.042
VIS	1.06 (1.04, 1.08)	<0.001	1.03 (1.01, 1.04)	0.006
Intraoperative fluid infusion; 10 mL	1.00 (1.00, 1.00)	<0.001	1.00 (1.00, 1.00)	0.320
Intraoperative red blood cell; unit	1.14 (1.09, 1.19)	<0.001	0.99 (0.88, 1.10)	0.825
Intraoperative blood loss; 10 mL	1.00 (1.00, 1.00)	<0.001	1.00 (1.00, 1.00)	0.977
Perioperative NSAID use	0.85 (0.73, 0.98)	0.030	0.72 (0.61, 0.85)	<0.001
Perioperative acetaminophen use	0.47 (0.41, 0.55)	<0.001	0.47 (0.40, 0.56)	<0.001
Perioperative steroid use	1.66 (1.43, 1.93)	<0.001	1.54 (1.26, 1.89)	<0.001
Perioperative antibiotics use	1.27 (1.00, 1.63)	0.052		
Perioperative antiepilepsy use	1.10 (0.70, 1.75)	0.674		

eGFR, estimated glomerular filtration rate; F, female; M, male; NSAID, Non-Steroidal Anti-Inflammatory Drug; VIS, Vasoactive-Inotropic Score.

**Table 4:** Long-term postoperative ALT elevation and 1-year mortality according to preoperative alanine aminotransferase concentrations in the matched cohort.

		**Matched Patients (n = 15,320)**	
**Long-term postoperative ALT elevation** (n = 10,016)		Odds Ratio (95% CI)	*p* value
Lower group	53/5008 (1.1%)	4.96 (3.65–6.74)	<0.001
Higher group	247/5008 (4.9%)		
**All-cause 1-year postoperative mortality** (n = 15,320)			
Lower group	334/7660 (4.4%)	1.25 (1.08, 1.45)	0.003
Higher group	412/7660 (5.4%)		

There were no ALT measurements after postoperative day 21 in 2986 patients in the matched cohort. Values are presented as n/N (%). Odds ratios were estimated from matched pairs. Analyses included only matched pairs with complete outcome data.

## Data Availability

The datasets generated and analyzed during the current study are available from the corresponding author on reasonable request.

## Supplementary Materials

The following supporting information can be downloaded at https://www.mdpi.com/article/10.3390/jcm15176824/s1. Figure S1. The associations of continuous variables with the probability of a marked postoperative ALT elevation. Figure S2. Rates of elevated preoperative alanine aminotransferase (ALT), marked postoperative ALT elevation, and 1-year mortality over time. Figure S3. Kernel density distributions and box plots showing the median and interquartile range of (A) preoperative ALT and (B) maximum ALT measured within 21 postoperative days in the lower (blue) and higher (orange) preoperative ALT groups in the propensity score-matched cohort. For visual clarity, the *y*-axis in Panel B was limited to 500 U/L. Table S1. Long-term postoperative ALT elevation and 1-year mortality according to preoperative ALT and marked postoperative ALT elevation in the overall and matched cohorts. Table S2. Baseline demographic, clinical, and preoperative characteristics of patients categorized into lower (male ≤ 34 U/L; female ≤ 22 U/L) and higher (male 35–82 U/L; female 23–66 U/L) preoperative ALT groups before and after propensity-score matching using preoperative covariates only.
