# Supplementary material for: Preoperative Alanine Aminotransferase and Postoperative ALT Elevation: A Retrospective Cohort Study
Source: J Clin Med. 2026 Sep 3;15(17):6824. doi: 10.3390/jcm15176824 (PMC13566529; doi:10.3390/jcm15176824)
Supplement: Supplementary file 1 [file jcm-15-06824-s001.zip › Figure S1 revised.pdf]

Figure S1. The associations of continuous variables with the probability of a marked postoperative ALT elevation.

Restricted cubic spline analyses of continuous variables in relation to the probability of marked postoperative ALT elevation. The curves show the nonlinear associations between continuous perioperative variables and the probability of marked postoperative ALT elevation, defined as a postoperative alanine aminotransferase (ALT) value >5 times the upper limit of normal within 21 days after surgery. Error bars represent 95% confidence intervals.

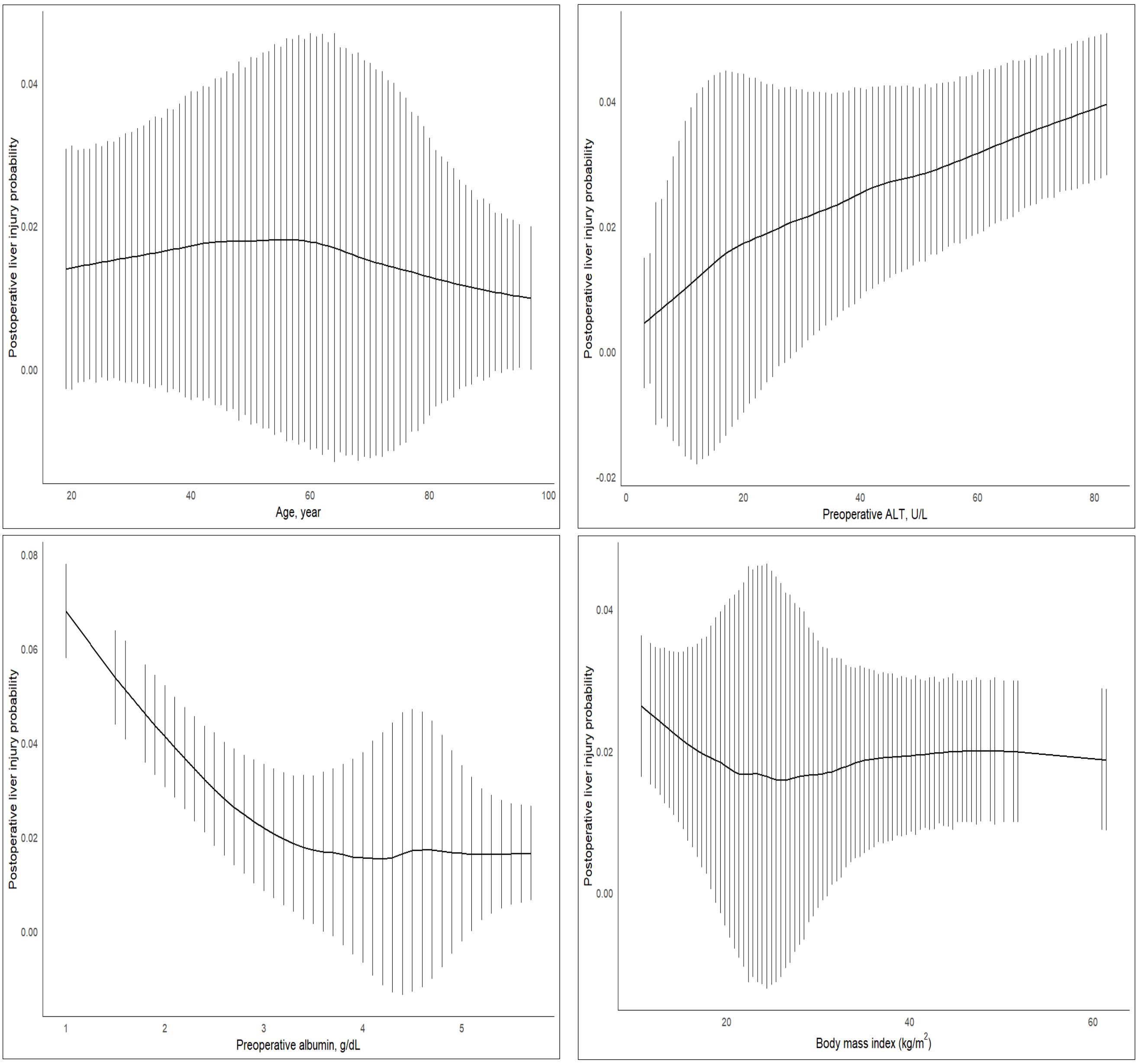

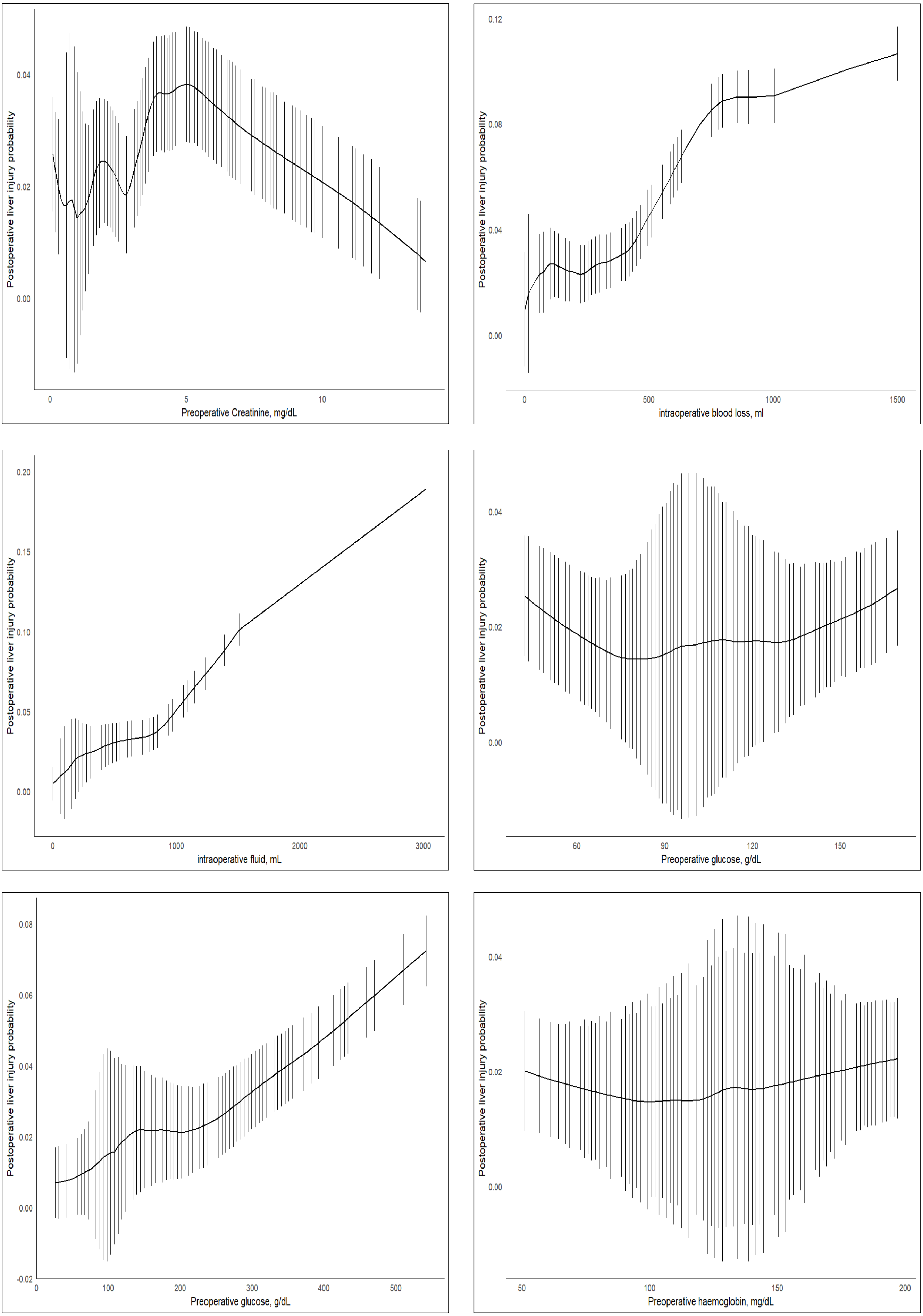
Postoperative liver injury probability
0.04
0.02
0.00
0
5
10
Preoperative Creatinine, mg/dL
Postoperative liver injury probability
0.12
0.08
0.04
0.00
0
500
1000
1500
intraoperative blood loss, ml
Postoperative liver injury probability
0.20
0.15
0.10
0.05
0.00
0
1000
2000
3000
intraoperative fluid, mL
Postoperative liver injury probability
0.04
0.02
0.00
60
90
120
150
Preoperative glucose, g/dL
Postoperative liver injury probability
0.08
0.06
0.04
0.02
0.00
-0.02
0
100
200
300
400
500
Preoperative glucose, g/dL
Postoperative liver injury probability
0.04
0.02
0.00
50
100
150
200
Preoperative haemoglobin, mg/dL

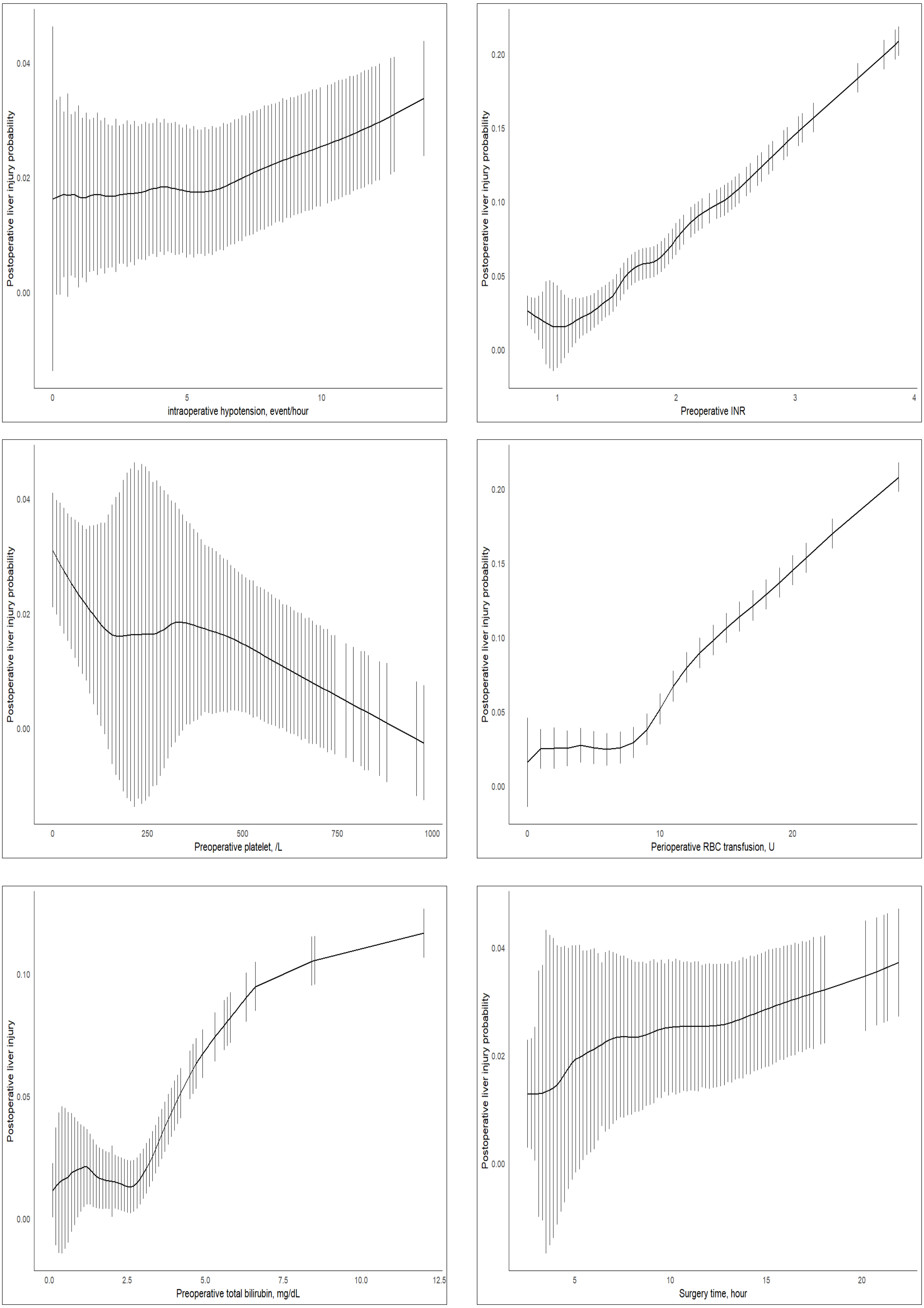

Postoperative liver injury probability
0.00
0.02
0.04
0
5
10
intraoperative hypotension, event/hour
Postoperative liver injury probability
0.00
0.05
0.10
0.15
0.20
1
2
3
4
Preoperative INR
Postoperative liver injury probability
0.00
0.02
0.04
0
250
500
750
1000
Preoperative platelet, /L
Postoperative liver injury probability
0.00
0.05
0.10
0.15
0.20
0
10
20
Perioperative RBC transfusion, U
Postoperative liver injury
0.00
0.05
0.10
0.0
2.5
5.0
7.5
10.0
12.5
Preoperative total bilirubin, mg/dL
Postoperative liver injury probability
0.00
0.02
0.04
5
10
15
20
Surgery time, hour

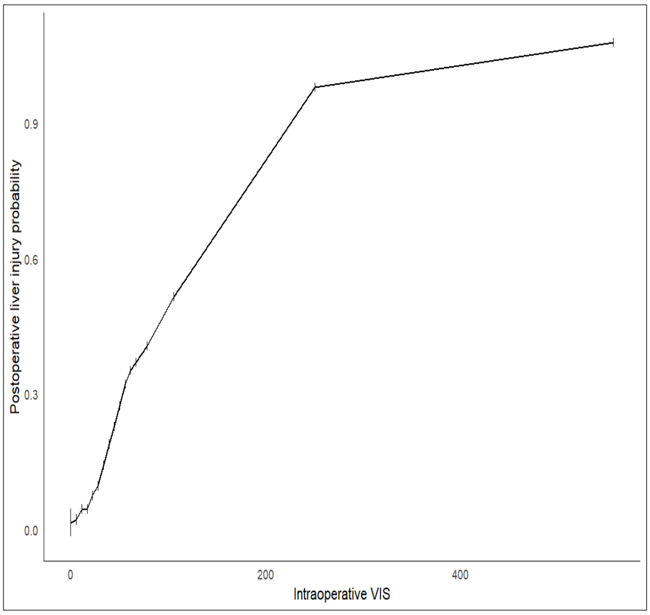

Postoperative liver injury probability
0.0
0.3
0.6
0.9
0
200
400
Intraoperative VIS
