# Supplementary material for: Preoperative Alanine Aminotransferase and Postoperative ALT Elevation: A Retrospective Cohort Study
Source: J Clin Med. 2026 Sep 3;15(17):6824. doi: 10.3390/jcm15176824 (PMC13566529; doi:10.3390/jcm15176824)
Supplement: Supplementary file 1 [file jcm-15-06824-s001.zip › Figure S2 revised.pdf]

Figure S2. Rates of elevated preoperative alanine aminotransferase (ALT), marked postoperative ALT elevation, and 1-year mortality over time.

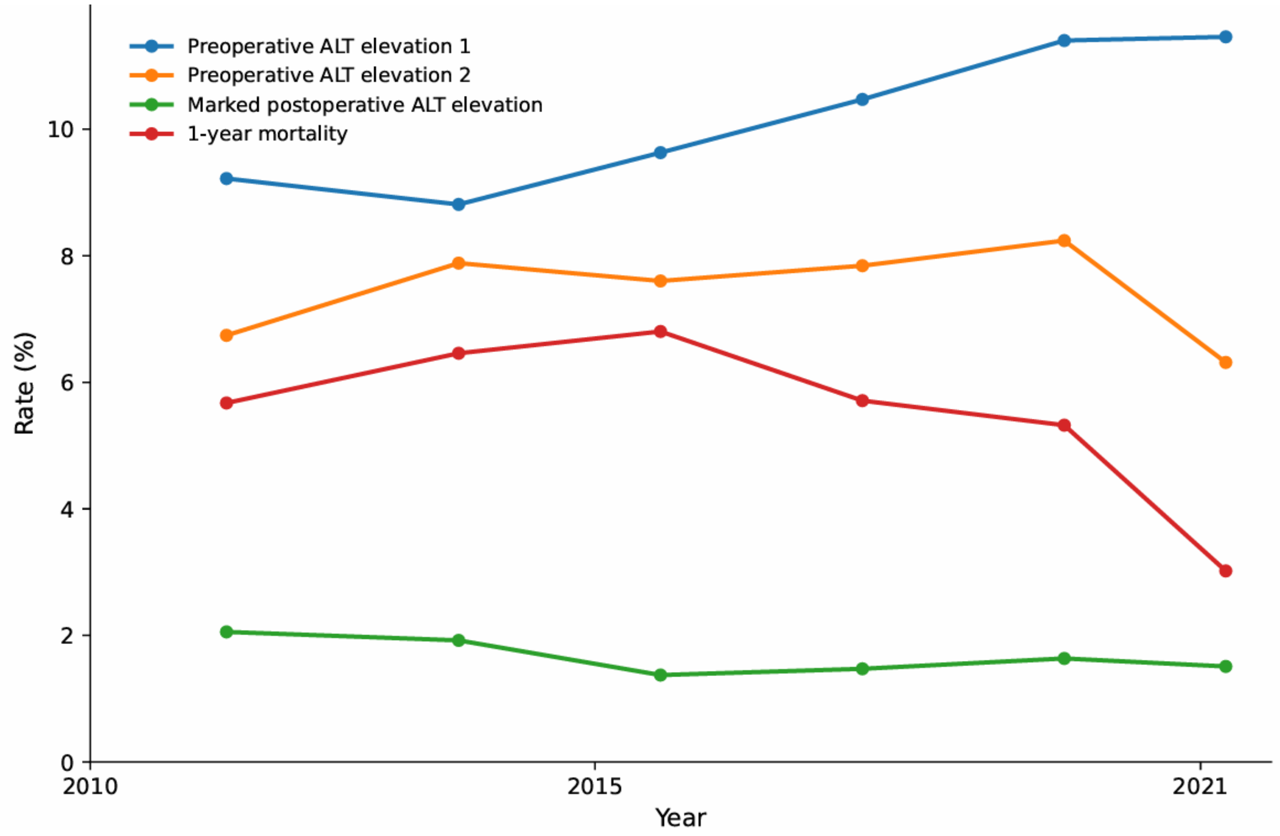

- Preoperative ALT elevation 1 means male 35–82 U/L; female 23–66 U/L based on proposed Asian reference intervals.
- Preoperative ALT elevation 2 means male 42–82 U/L; female 34–66 U/L based on the institutional reference intervals.
- Marked postoperative ALT elevation defined as any postoperative serum ALT value measured within 21 days after surgery that exceeded five times the upper limit of normal.
