# Supplementary material for: Preoperative Alanine Aminotransferase and Postoperative ALT Elevation: A Retrospective Cohort Study
Source: J Clin Med. 2026 Sep 3;15(17):6824. doi: 10.3390/jcm15176824 (PMC13566529; doi:10.3390/jcm15176824)

Figure S3. Kernel density distributions and box plots showing the median and interquartile range of (A) preoperative ALT and (B) maximum ALT measured within 21 postoperative days in the Lower (blue) and Higher (orange) preoperative ALT groups in the propensity score-matched cohort. For visual clarity, the y-axis in Panel B was limited to 500 U/L.

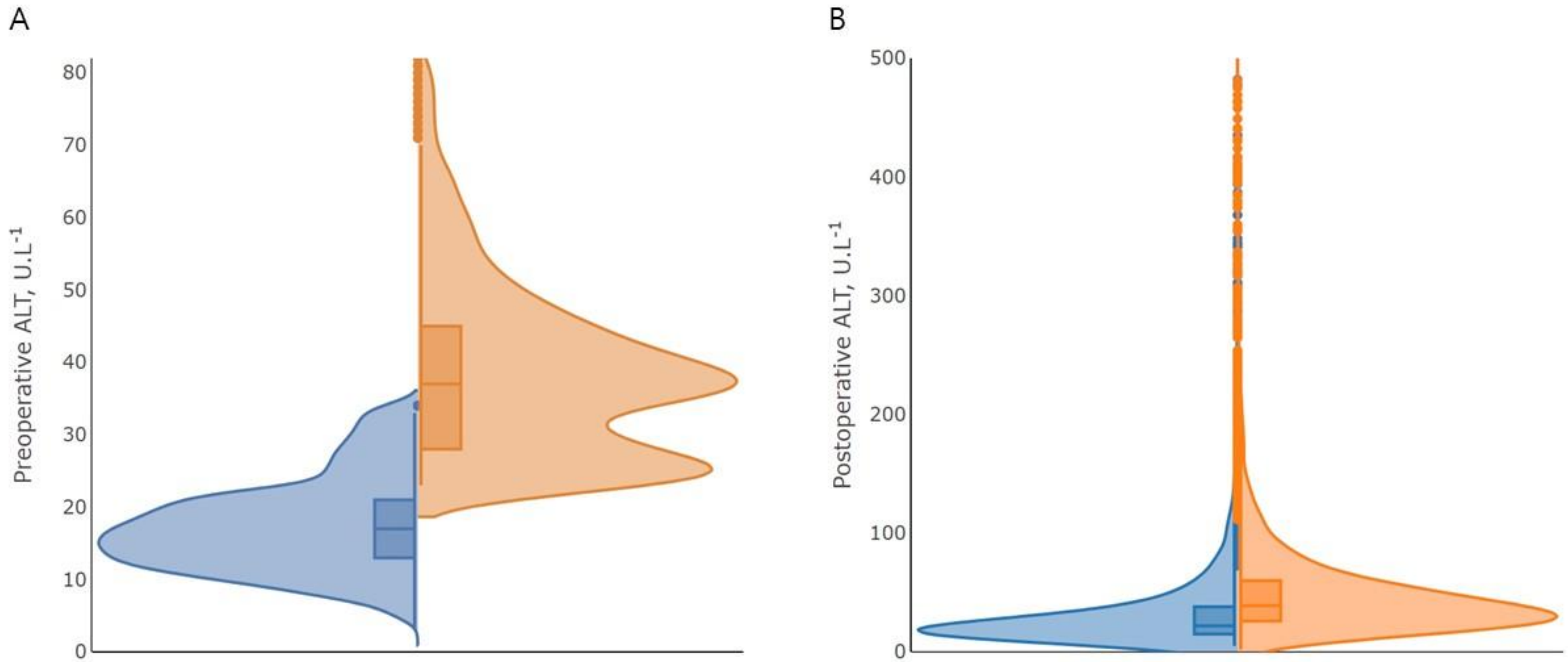

Supplement: Supplementary file 1 [file jcm-15-06824-s001.zip › Figure S3 revised.pdf]
