# Supplementary material for: Preoperative Alanine Aminotransferase and Postoperative ALT Elevation: A Retrospective Cohort Study
Source: J Clin Med. 2026 Sep 3;15(17):6824. doi: 10.3390/jcm15176824 (PMC13566529; doi:10.3390/jcm15176824)
Supplement: Supplementary file 1 [file jcm-15-06824-s001.zip › Table S1 revised.pdf]

Table S1. Long-term postoperative ALT elevation and 1-year mortality according to preoperative ALT and marked postoperative ALT elevation in the overall and matched cohorts.

**Overall patients (n=43892)**						
**Postoperative status**	**Long-term ALT Outcome (n=37075)**					
	**Normal**	**<2 × ULN**	**2–5 × ULN**	**5–15 × ULN**	**>15 × ULN**	**1-yr death**
Normal (n=30,758; 70.1%)	24829	256	238	4	0	1392
<2 × ULN (n=9147; 20.8%)	7680	249	200	0	0	596
2–5 × ULN (n=3254; 7.4%)	2786	87	83	1	0	287
5–15 × ULN (n=621; 1.4%)	537	10	21	0	0	72
>15 × ULN (n=112; 0.3%)	87	4	3	0	0	33

	**Long-term ALT >X2 ULN**			**1-yr mortality**		
	Incidence	(OR, 95% CI)	P value	Incidence	(OR, 95% CI)	P value
Preoperative Higher ALT	313/6262 vs 237/30813	6.79 (5.72, 8.05)	<.001	418/7740 vs 1962/36152	0.99 (0.89–1.11)	.925
Marked postoperative ALT elevation	24/550 vs 638/36525	2.57 (1.69, 3.89)	<.001	105/733 vs 2275/43159	3.00 (2.43–3.71)	<.001

**Matched patients (n=15320)**						
**Postoperative status**	**Long-term ALT Outcome (n=12334)**					
	**Normal**	**<2 × ULN**	**2–5 × ULN**	**5–15 × ULN**	**>15 × ULN**	**1-yr death**
Normal (n=9473; 61.8%)	7032	104	136	2	0	344
<2 × ULN (n=4037; 26.4%)	3104	166	161	0	0	231
2–5 × ULN (n=1485; 9.7%)	1214	60	60	1	0	129
5–15 × ULN (n=287; 1.9%)	242	5	14	0	0	34
>15 × ULN (n=38; 0.3%)	30	1	2	0	0	8

	**Long-term ALT >X2 ULN**			**1-yr mortality**		
	Incidence	(OR, 95% CI)	P value	Incidence	(OR, 95% CI)	P value
Preoperative Higher ALT	311/6206 vs 65/6128	4.92 (3.76, 6.44)	<.001	412/7660 vs 334/7660	1.25 (1.08, 1.45)	.003
Marked postoperative ALT elevation	16/294 vs 360/12040	1.87 (1.12, 3.12)	.030	42/325 vs 704/14995	3.01 (2.16, 4.20)	<.001

The maximal postoperative ALT value was classified according to the American College of Gastroenterology guidelines based on multiples of the upper limit of normal (ULN): <2 × ULN, 2–5 × ULN, 5–15 × ULN, and >15 × ULN. ALT values ≤ULN were considered normal. The institutional ULNs for ALT were 41 U/L for men and 33 U/L for women.

*ALT measurements between postoperative days 22 and 365 were unavailable in 6,817 patients in the overall cohort and 2,986 patients in the matched cohort.

Values are n/N (%). ORs are unadjusted. All patients with available data for each outcome were included; matched analyses were not restricted to pairs with complete follow-up data for both members. Long-term postoperative ALT elevation was defined as an increase in ALT from the preoperative value to >2× the institutional ULN at the last available measurement between postoperative days 22 and 365.
