# Supplementary material for: Preoperative Alanine Aminotransferase and Postoperative ALT Elevation: A Retrospective Cohort Study
Source: J Clin Med. 2026 Sep 3;15(17):6824. doi: 10.3390/jcm15176824 (PMC13566529; doi:10.3390/jcm15176824)
Supplement: Supplementary file 1 [file jcm-15-06824-s001.zip › Table S2 revised.pdf]

Table S2. Baseline demographic, clinical, and preoperative characteristics of patients categorized into Lower (male ≤34 U/L; female ≤22 U/L) and Higher (male 35–82 U/L; female 23–66 U/L) preoperative ALT groups before and after propensity-score matching using preoperative covariates only.

Variables	All patients			Matched patients		
	Lower n = 36,152	Higher n = 7740	SMD	Lower n = 7707	Higher n = 7707	SMD
Department of surgery						
General surgery	10012 (27.7%)	1635 (21.1%)	.153	1615 (21.0%)	1632 (21.2%)	.005
Orthopedics	3791 (10.5%)	1072 (13.9%)	.103	1095 (14.2%)	1061 (13.8%)	.013
Neurosurgery	7728 (21.4%)	2364 (30.5%)	.210	2382 (30.9%)	2353 (30.5%)	.008
Gynecology	1280 (3.5%)	308 (4.0%)	.023	312 (4.0%)	305 (4.0%)	.005
Urology	5291 (14.6%)	885 (11.4%)	.095	871 (11.3%)	885 (11.5%)	.006
Thoracic surgery-pulmonary	5369 (14.9%)	857 (11.1%)	.113	836 (10.8%)	857 (11.1%)	.009
Others	2681 (7.4%)	619 (8.0%)	.022	596 (7.7%)	614 (8.0%)	.009
Cancer surgery	28891 (79.9%)	5672 (73.3%)	.157	5641 (73.2%)	5652 (73.3%)	.003
Emergency	1761 (4.9%)	480 (6.2%)	.058	520 (6.7%)	476 (6.2%)	.023
Date of surgery						
2010.1-2012.6	7155 (19.8%)	1359 (17.6%)	.057	1354 (17.6%)	1359 (17.6%)	.002
2012.7 - 2014.6	5030 (13.9%)	1008 (13.0%)	.026	1024 (13.3%)	1007 (13.1%)	.007
2014.7 - 2016.6	5183 (14.3%)	1079 (13.9%)	.011	1025 (13.3%)	1076 (14.0%)	.019
2016.7 - 2018.6	5937 (16.4%)	1331 (17.2%)	.021	1316 (17.1%)	1321 (17.1%)	.002
2018.7 - 2020.6	6583 (18.2%)	1609 (20.8%)	.065	1618 (21.0%)	1599 (20.7%)	.006
2020.7 - 2021.8	6264 (17.3%)	1354 (17.5%)	.004	1370 (17.8%)	1345 (17.5%)	.009
Age; year	58.39 (14.00)	55.81 (13.13)	.190	55.92 (14.51)	55.85 (13.11)	.005
Sex, Male	14484 (40.1%)	4066 (52.5%)	.252	4111 (53.3%)	4040 (52.4%)	.018
Body mass index; kg/m^2	24.07 (3.39)	25.75 (3.97)	.453	25.68 (3.90)	25.69 (3.85)	.003
ASA physical status						
1	8458 (23.4%)	1638 (21.2%)	.054	1622 (21.0%)	1636 (21.2%)	.004
2	23270 (64.4%)	5267 (68.0%)	.078	5208 (67.6%)	5243 (68.0%)	.010
3	4163 (11.5%)	788 (10.2%)	.043	824 (10.7%)	781 (10.1%)	.018
4	240 (0.7%)	46 (0.6%)	.009	48 (0.6%)	46 (0.6%)	.003
5	21 (0.1%)	1 (0.0%)	.024	5 (0.1%)	1 (0.0%)	.026
Liver disease	1805 (5.0%)	543 (7.0%)	.085	519 (6.8%)	535 (7.0%)	.008
Hypertension	10747 (29.7%)	2493 (32.2%)	.054	2500 (32.4%)	2477 (32.1%)	.006
Stroke	891 (2.5%)	185 (2.4%)	.005	189 (2.5%)	184 (2.4%)	.004
Heart failure	109 (0.3%)	15 (0.2%)	.022	14 (0.2%)	15 (0.2%)	.003
Diabetes mellitus	4887 (13.5%)	1108 (14.3%)	.023	1092 (14.2%)	1100 (14.3%)	.003
Coronary artery disease	1144 (3.2%)	189 (2.4%)	.044	181 (2.3%)	188 (2.4%)	.006
Chronic obstructive pulmonary disease	1026 (2.8%)	124 (1.6%)	.084	140 (1.8%)	124 (1.6%)	.016
Current alcohol consumption	7577 (21.0%)	1565 (20.2%)	.018	1577 (20.5%)	1558 (20.2%)	.006
Current smoking	3162 (8.7%)	726 (9.4%)	.022	736 (9.5%)	723 (9.4%)	.006
Hyperlipidemia	2091 (5.8%)	596 (7.7%)	.076	564 (7.3%)	590 (7.7%)	.013
Pre-operative laboratory						
Hemoglobin; g/L	132.65 (17.97)	136.96 (17.70)	.241	136.35 (16.71)	136.93 (17.69)	.034
Albumin; g/dL	4.34 (0.42)	4.40 (0.43)	.133	4.39 (0.39)	4.40 (0.43)	.036
eGFR; mL/min/1.73m^2	100.01 (11.64)	102.44 (10.91)	.215	102.41 (11.60)	102.39 (10.88)	.002
Total bilirubin; mg/dL	0.57 (0.35)	0.58 (0.37)	.007	0.58 (0.36)	0.58 (0.37)	.005

International normalized ratio	0.99 (0.10)	0.97 (0.09)	.176	0.97 (0.08)	0.97 (0.09)	.018
Platelet	241.83 (71.38)	244.05 (73.50)	.031	243.82 (66.81)	243.86 (73.46)	.001
Fasting glucose, mg/dL	112.18 (29.59)	116.46 (32.23)	.138	116.83 (34.30)	116.39 (32.14)	.013
Surgical risk						
Low	3842 (10.6%)	1013 (13.1%)	.076	1032 (13.4%)	1002 (13.0%)	.012
Intermediate	30124 (83.3%)	6450 (83.3%)	.000	6409 (83.2%)	6428 (83.4%)	.007
High	2186 (6.0%)	277 (3.6%)	.115	266 (3.5%)	277 (3.6%)	.008
Preoperative medication use						
NSAID use	1286 (3.6%)	349 (4.5%)	.048	378 (4.9%)	347 (4.5%)	.019
Acetaminophen use	1745 (4.8%)	440 (5.7%)	.038	470 (6.1%)	436 (5.7%)	.019
Steroid use	5061 (14.0%)	1498 (19.4%)	.144	1495 (19.4%)	1486 (19.3%)	.003
Antibiotics use	475 (1.3%)	126 (1.6%)	.026	136 (1.8%)	126 (1.6%)	.010
Antiepileptic use	705 (2.0%)	172 (2.2%)	.019	184 (2.4%)	171 (2.2%)	.011

Data are presented as mean (standard deviation) or number (%). SMD, standardized mean difference; ALT, alanine aminotransferase; ASA, American Society of Anesthesiologists; eGFR, estimated glomerular filtration rate; NSAID, non-steroidal anti-inflammatory drug.
